# Intergenerational support, activities of daily living, and the interaction on psychological distress in older adults

**DOI:** 10.3389/fpsyg.2024.1454662

**Published:** 2024-12-05

**Authors:** Juan Zheng, Jianqiang Xu, Yuhang Wu, Shuo Xu, Yang Gao

**Affiliations:** School of Management, Xuzhou Medical University, Xuzhou, Jiangsu, China

**Keywords:** intergenerational support, activities of daily living, psychological distress, older adults, interaction

## Abstract

**Objectives:**

The purpose of this study is to examine the effects of intergenerational support and activities of daily living (ADL) on psychological distress in older adults, and to analyse the effects of the interaction between ADL and intergenerational support on psychological distress in older adults.

**Methods:**

A probability sample of 1,065 older adults aged 60 and above was conducted in Xuzhou, China, from 18 June to 26 August 2023. ADL was assessed using the ADL scale combined with the Barthel Index; Intergenerational support was measured using the Intergenerational Support Scale; and psychological distress was measured using the Kessler 10 scale. An ordered multichotomous logistic regression model was constructed to analyse the factors influencing psychological distress in the elderly. The interaction model was constructed by one-way analysis of variance to further analyse the effect of the interaction between financial support, instrumental support and emotional support and ADL on psychological distress in the elderly.

**Results:**

Elderly living in rural areas, with an annual household income of less than 30,000 yuan, who engaged in manual labor before retirement, who did not receive financial support and who received instrumental support were more likely to experience psychological distress, and the higher the degree of restriction in ADL, the higher the risk of psychological distress. A significant interaction was observed between ADL and financial support (*F* = 221.570, *p* < 0.05), as well as between ADL and emotional support (*F* = 399.805, *p* < 0.01). Additionally, a significant interaction was found among ADL, financial support, and instrumental support (*F* = 227.187, *p* < 0.05).

**Conclusion:**

(1) A significant association between place of residence, annual household income, occupation, financial support, instrumental support, ADL, and psychological distress in older adults; (2) When the level of ADL was moderate, the risk of psychological distress in older adults who received emotional support was significantly lower than that of older people who did not receive emotional intergenerational support; When the level of ADL was completely independent, financial support was beneficial in reducing psychological distress in the elderly; (3) the risk of psychological distress in the elderly who received instrumental support increased with the severity of ADL limitations, regardless of whether they received additional financial support.

## 1 Introduction

The development of China’s social economy and the adjustment of its birth policy have led to an increase in the elderly population. As the world’s most populous country, China’s ageing population is growing at a faster rate than that of other countries (Wei et al., 2019). According to the results of the seventh national census, people aged 60 and above account for 18.7% of China’s total population (National Bureau of Statistics, 2020). It is estimated that by 2030, the elderly population aged 60 and above will reach 25.3% of the total population (Bai et al., 2021). The elderly are a major consumer of medical resources and are also a high-risk group for mental health. Psychological distress was an unpleasant emotional experience caused by multiple factors involving psychological (cognitive, behavioral, and emotional), social, and spiritual changes, in which the patient progressed from common fears, sadness, and vulnerability to anxiety, depression, fear, and social independence, whereas symptoms of depression, anxiety, and stress were collectively referred to as psychological distress (Xiao et al., 2022). These mental health disorders can adversely affect the physical health of the elderly and can increase the risk of suicide and death among the elderly (Chen et al., 2014). The global social issue of achieving healthy ageing, reducing the risk of psychological distress among the elderly and improving their mental health has become a pressing concern (Behr et al., 2023).

Notably, psychological health of the elderly is related to numerous factors, and from the perspective of the external environment, based on several previous studies, place of residence, income level, and occupational nature are a few non-negligible variables closely related to psychological distress among the elderly, and higher levels of psychological distress are associated with living in rural areas (Janzen and Hellsten, 2021), having a low level of average annual income (Cheng and Silverberg, 2019), and engaging in manual labor prior to retirement (Honkaniemi et al., 2020). As a result of regional development levels and geographic differences, older adults in urban areas and with higher levels of household income are more likely to have access to quality healthcare resources and recreational modalities or facilities that generate positive, optimistic moods and are effective in avoiding negative or negative mental health risk factors.

From the perspective of family support, previous research has shown that family support and the nature or type of support can affect the health and wellbeing of elders, and suggests that such support may reduce the risk of poor health that elders face (Shah et al., 2022). A number of studies have demonstrated that intergenerational support acts as a protective factor against psychological distress in older people (Buber and Engelhardt, 2008) and can effectively regulate depressive symptoms (Zhang J. et al., 2022; Wang et al., 2022). Intergenerational support is defined as the economic, care, and emotional interactions between parents and children within the family (Zheng et al., 2022). In previous studies, intergenerational support has often been measured through three dimensions, including financial support, emotional support, and instrumental support. The direction of intergenerational support can be divided into three categories: forward (elders providing support to the younger generation), backward (the younger generation providing support to the elders), and mutual support (Zheng et al., 2022; Teerawichitchainan et al., 2015). Financial support is the transfer of economic amounts between generations of a family, mainly in the form of money or goods to meet the living needs of children or parents (Rittirong et al., 2014; Gualano et al., 2018). Some studies have demonstrated that financial support can enhance the life satisfaction and happiness of the elderly (Huang and Fu, 2021), particularly for the elderly with functional limitations, whose care needs and medical expenses are higher (Zhou et al., 2023). Financial support from children can assist them in avoiding the dual dilemma of disability and poverty due to financial constraints (Xia et al., 2022). Nevertheless, some scholars have identified in their research that excessive intergenerational financial support can impair the self-esteem and self-confidence of the elderly, leading to psychological issues (Zheng et al., 2022; Fingerman et al., 2013). Emotional support encompasses communication, trust and companionship and reflects the closeness and stability of intergenerational relationships (Sun et al., 2022). A study has demonstrated that as people age, their capacity to complete daily tasks independently diminishes, and their social interaction patterns evolve. They are more inclined to maintain stable relationships with family members with whom they have close emotional ties (Lai et al., 2019; Antonucci et al., 2014). Emotional support serves to reassure the elderly that they are cared for by their children, and can significantly enhance their sense of wellbeing (Zheng et al., 2022), maintain their mental health and reduce the incidence of depression (Roh et al., 2015; Koelmel et al., 2017). However, other studies have indicated that excessive or insufficient emotional support from children does not have a significant effect on the maintenance of the mental health of the elderly (Teixeira et al., 2016). Instrumental support is defined as assistance in the form of practical help, including nursing, care and household chores (Sun et al., 2022; Kent et al., 2020). In some studies, instrumental support has been found to be the most common form of support provided by adult children to their parents in filial piety cultures (Zhou et al., 2023; Kalmijn, 2018). This form of support has been shown to have a positive impact on the wellbeing of the elderly, reducing the level of depressive symptoms and psychological distress caused by illness or disability (Koelmel et al., 2017). Furthermore, it helps to maintain physical health and improve life satisfaction (Huang and Fu, 2021; Tang et al., 2022).

Conversely, findings from other studies indicate that the impact of instrumental support on the mental health of the elderly is not always positive, and may even be detrimental (Van de Velde et al., 2010). In particular, for the elderly who are disabled, long-term family care can cause significant physical and mental stress (Chen et al., 2018), increasing their sense of inferiority and burden (Schroepfer and Noh, 2010; Fu et al., 2024). Furthermore, long-term daily care may give rise to intergenerational conflicts, which may result in a reduction in the willingness of children to care for their parents and an increased risk of psychological distress in the elderly, which has a detrimental effect on their mental health (Chen et al., 2018; Dong et al., 2017).

Moreover, from the perspective of the elderly’s own activity functioning, the emergence of a negative mental health state in the elderly can be linked to the lack of ability to perform basic daily activities such as dressing, washing or shopping. Activities of daily living (ADL) refer to the most basic and common activities that people repeat on a daily basis in order to maintain and adapt to their living environment (McLachlan and Gale, 2018). In addition, ADL is also an indicator of a person’s functional status and has a significant impact on their mental state. Previous studies have demonstrated that when ADL is limited, the elderly are unable to complete daily activities independently, become dependent on others for assistance for an extended period, lack confidence in solving problems, and experience psychological distress, including depression, anxiety, and stress (Nie et al., 2020).

The psychological distress of the elderly is influenced by intergenerational support from children and ADL. It would be beneficial to conduct further research to ascertain whether there is an interaction between the two. The existing literature indicates that scholars have focused their research on the relationship between intergenerational support from children and depression in the elderly, the relationship between ADL and depression in the elderly, and the relationship between dysfunction or disability and psychological distress in the elderly. A limited number of scholars have investigated the interaction between different levels of ADL and intergenerational support from children on the psychological distress of the elderly. The objective of this study is to ascertain the impact of ADL and intergenerational support on the psychological distress of the elderly, and to investigate whether the interaction between ADL and intergenerational support affects the psychological distress of the elderly. The investigation of this potential relationship will not only facilitate the improvement of the mental health of the elderly and the acceleration of healthy ageing, but also provide direction and information support for the government, society and family members in the prevention of mental health disorders in the elderly.

## 2 Methods

### 2.1 Study design and respondents

The study was conducted between 18 June and 26 August 2023. Xuzhou City was divided into 10 strata according to the division of districts or counties by stratified random sampling. At least 85 elderly individuals aged 60 or above were randomly selected from each district or county in each stratum. Inclusion criteria were as follows: (1) aged 60 or above; (2) residing in the survey site for a period of 6 months or more; (3) possessing normal language expression and communication skills; (4) willing to participate in the survey. Exclusion criteria were as follows: (1) elderly individuals with language barriers; (2) elderly individuals with poor compliance; (3) migrant population from other provinces. To encourage a greater number of respondents to participate in the survey, the research team provided food and some daily necessities, thereby increasing the motivation of the respondents. Prior to the commencement of the survey, the investigators conducted face-to-face communication with the respondents. All respondents were informed of the purpose, significance and progress of the study and signed an informed consent form. At the same time, respondents were also informed that they had the right to withdraw from the study at any time. After the exclusion of questionnaires with missing key information and dropouts, a total of 1,065 respondents aged 60 and above completed the questionnaire. The questionnaire included the following sections: (1) socio-demographic characteristics; (2) intergenerational support; (3) ADL level; (4) psychological distress. The study was approved by the Ethics Committee of Xuzhou Medical University (Ethics approval no.: XZHMU-2022085).

### 2.2 Socio-demographic characteristics

The socio-demographic characteristics collected include gender (male, female), age (60–69 years, 70–79 years, ≥80 years), area of residence (urban, rural), marital status (single, married, others), living arrangement (alone, non-alone), educational level (primary school or below, middle school education, bachelor’s degree or above), annual household income (≤30,000 yuan, ≥30,000 yuan), nature of occupation before retirement (mental work, physical work).

### 2.3 Questionnaires

#### 2.3.1 Intergenerational support

The intergenerational support is evaluated from three dimensions: financial support, instrumental support and emotional support (Li et al., 2022). The evaluation items for financial support include: (a) Did your children provide monthly financial support in the past year (specifically necessary living expenses as opposed to extra monetary purchases such as traveling or purchasing luxury items etc.)? (b) Was the financial support provided by your children regular? The answers to the questions were coded separately, with a direct code of 0 for no monthly financial support from children and a code of 1 for otherwise. The items for assessing instrumental support included: (a) Did your children provide care when you need help with daily living or when you are sick or ill? (b) Were your children able to help you solve problems? The answers to the questions were coded separately, with a direct code of 0 if the child did not provide care, and a code of 1 otherwise. The items for assessing emotional support were: (a) Whether your children were willing to communicate with you? (b) Did your children regularly engage in affectionate behaviors with you (e.g., hugging)? The answers to the questions were coded separately, with a direct code of 0 if the child was not willing to communicate, and a code of 1 otherwise. These questions were adapted from the China Health and Longevity Longitudinal Study (Wang et al., 2014). The Cronbach’s *α* coefficient for this scale in this study was 0.764.

#### 2.3.2 Activities of daily living

The Barthel Index was employed to assess the ability to perform activities of daily living (ADL) in this study (Katz et al., 1970). The Index comprises 10 items: eating, washing, grooming, dressing, bowel control, bladder control, toileting, bed transfer, walking, and climbing stairs. The total score is 100, with higher scores indicating greater independence in daily life (Xu et al., 2019). The score can be divided into four categories. A score below 40 indicates severe impairment, with the individual completely unable to care for themselves and dependent on the help of others. A score between 41 and 60 indicates moderate impairment, with the individual requiring assistance from others. A score between 61 and 99 indicates mild impairment, with the individual being able to care for themselves to a certain extent. A score of 100 indicates complete independence in ADL (Fong, 2019). Previous studies have demonstrated that the ADL scale has high reliability and validity among the elderly population in China (Feng et al., 2013). The Cronbach’s *α* coefficient for this scale in this study was 0.955.

#### 2.3.3 Psychological distress

The Kessler 10 (K10) scale is employed to assess psychological distress. This scale comprises 10 items, each of which enquires about the frequency of symptoms experienced over the past 30 days. The scale is designed to measure psychological distress by examining symptoms of depression (5 items) and anxiety (5 items) (Kessler et al., 1994). Respondents are asked to answer questions on a 5-point Likert scale, with each question ranging from 1 (almost never) to 5 (always). The scores for each item are added to calculate the score. The final score for the K10 scale ranges from 10 to 50 points, with higher scores indicating higher levels of psychological distress (Andrews and Slade, 2001). Previous studies have classified the K10 scale into four categories: A score of 10–19 points indicates a low risk of psychological distress, 20–24 points indicate mild symptoms, 25–29 points indicate moderate symptoms, and 30–50 points indicate a very high risk of mental disorders (Hutchesson et al., 2021). The K10 scale has been widely used in academic and clinical research and has demonstrated strong reliability and validity in the general population (Kessler et al., 1994; Xu et al., 2017). The Cronbach’s α of the entire scale in this study was 0.931.

#### 2.3.4 Statistical analysis

The statistical analysis was conducted using SPSS 22.0 software (IBM SPSS Statistics GradPack Premium). Descriptive analysis was employed to examine the basic characteristics of the data, while the *χ*^2^ test was utilized to assess the levels of psychological distress between groups of elderly individuals with varying characteristics. A logistic regression model with ordered multiple categories was established to analyse the factors influencing the risk of psychological distress in the elderly. The Hosmer-Lemeshow goodness-of-fit test and the area under the receiver operating characteristic (ROC) curve were employed to assess the goodness-of-fit and predictive accuracy of the model, respectively. The interaction between activities of daily living (ADL) and intergenerational support on psychological distress was analysed using a one-way analysis of variance. In the context of the interaction analysis, a *p* < 0.05 for the interaction term indicates a statistically significant interaction. The normality of the data was tested by comparing the absolute values of skewness and kurtosis. The skewness of psychological distress was 1.602, while the kurtosis was 2.644. The skewness of ADL was 2.353, with a kurtosis of 5.738. The skewness of financial support was −0.624, with a kurtosis of 1.247. The skewness of emotional support was −0.653, with a kurtosis of 0. The absolute values of the skewness of these variables were within 2 and the kurtosis was within 7, indicating that the data approximately obeyed a normal distribution. The homogeneity of variance was verified by means of the Levene test. A value of *p* > 0.05 indicates that the variance of the population represented by the sample is homogeneous. The significance of ADL was found to be *p* = 0.291, that of financial support *p* = 0.156, that of emotional support *p* = 0.274, and that of instrumental support *p* = 0.292. Consequently, it can be posited that the population represented by the sample exhibits equal variance.

## 3 Results

### 3.1 Descriptive analysis

Table 1 presents the univariate analysis of the socio-demographic characteristics and the risk of psychological distress in the elderly. A total of 1,065 individuals completed the questionnaire, of whom 848 (79.6%) exhibited a low level of risk of psychological distress, 146 (13.7%) exhibited a slight risk, 39 (3.6%) exhibited a moderate risk, and 33 (3.1%) exhibited a high risk. 522 (49.0%) were male; 466 (43.8%) were aged 60–69, 425 (39.9%) were aged 70–79, and 174 (16.3%) were aged 80 and above. Of the total sample, 381 (35.8%) were urban residents, 32 (3.0%) were single, 835 (78.4%) were married, and 198 (18.6%) were in other categories. A total of 157 individuals (14.7%) reside alone, while 676 individuals (30.5%) have attained a primary school education or below, 355 individuals (33.3%) have completed secondary school, and 34 individuals (3.2%) have obtained a bachelor’s degree or above. The mean annual household income was below 30,000 yuan for 176 individuals (16.5%); 244 individuals (22.9%) had engaged in mental work prior to retirement; 396 individuals (37.2%), 257 individuals (24.1%), and 551 individuals (51. 7%) of the respondents did not receive financial, emotional, or instrumental support from their children in the past year. A total of 895 people (84.1%) had an ADL level of complete self-care, 114 people (10.7%) had a mild disability, 35 people (3.3%) had a moderate disability, and 21 people (1.9%) had a severe disability. 7% of respondents indicated that they had not received financial, emotional, or instrumental support from their children in the past year. In contrast, 895 respondents (84.1%) exhibited an ADL level of complete self-care, while 114 respondents (10.7%) exhibited mild impairment, 35 respondents (3.3%) exhibited moderate impairment, and 21 respondents (1.9%) exhibited severe impairment. The results of the univariate analysis indicated that age (*χ*^2^ = 19.817, *p* < 0.05), place of residence (*χ*^2^ = 10.905, *p* < 0.05), marital status (*χ*^2^ = 17.272, *p* < 0.05), annual family income (*χ*^2^ = 10.431, *p* < 0.05), occupation (*χ*^2^ = 9.376, *p* < 0.05), emotional support (*χ*^2^ = 15.590, *p* < 0.05), instrumental support (*χ*^2^ = 17.459, *p* < 0.05), and ADL level (*χ*^2^ = 208.504, *p* < 0.001) were statistically significant (*p* < 0.05) for the risk of psychological distress in the elderly.

**Table 1:** Univariate analysis of the risk of psychological distress in the elderly.

Variables	Psychological distress						
	*N* (%)	Low	Mild	Moderate	High	*χ*^2^	*P*
Characteristics							
Sex						4.638	0.212
Male	522 (49)	427 (81.8)	60 (11.5)	20 (3.8)	15 (2.9)		
Female	543 (51)	421 (77.5)	86 (15.8)	18 (3.3)	18 (3.3)		
Age						19.817	0.003
60–69 years	466 (43.8)	375 (80.5)	65 (13.9)	14 (3.0)	12 (2.6)		
70–79 years	425 (39.9)	347 (81.6)	55 (12.9)	16 (3.8)	7 (1.6)		
≥80 years	174 (16.3)	126 (72.4)	26 (14.9)	8 (4.6)	14 (8.0)		
Area of residence						10.905	0.012
Urban	381 (35.8)	321 (84.3)	38 (10.0)	15 (3.9)	7 (1.8)		
Rural	684 (64.2)	527 (77.0)	108 (15.8)	23 (3.4)	26 (3.8)		
Marital status						17.272	0.008
Single	32 (3.0)	26 (81.3)	4 (12.5)	1 (3.1)	1 (3.1)		
Married	835 (78.4)	683 (81.8)	105 (12.6)	28 (3.4)	19 (2.3)		
Others	198 (18.6)	139 (70.2)	37 (18.7)	9 (4.5)	13 (6.6)		
Living situation						7.488	0.058
Lives alone	157 (14.7)	117 (74.5)	23 (14.6)	7 (4.5)	10 (6.4)		
Living with others	908 (85.3)	731 (80.5)	123 (13.5)	31 (3.4)	23 (2.5)		
Educational level						5.698	0.494
Primary school or below	676 (30.5)	526 (77.8)	104 (15.4)	25 (3.7)	21 (3.1)		
Middle school education	355 (33.3)	295 (83.1)	38 (10.7)	11 (3.1)	11 (3.1)		
Bachelor’s degree or above	34 (3.2)	27 (79.4)	4 (11.8)	2 (5.9)	1 (2.9)		
Annual household income						10.431	0.015
<30000Yuan	176 (16.5)	131 (74.4)	36 (20.5)	7 (4.0)	2 (1.1)		
≥30000Yuan	889 (83.5)	717 (80.7)	110 (12.4)	31 (3.5)	31 (3.5)		
Occupation before retirement						9.376	0.025
Mental work	244 (22.9)	208 (85.2)	29 (11.9)	5 (2.0)	2 (0.8)		
Manual labor	821 (77.1)	640 (78.0)	117 (14.3)	33 (4.0)	31 (3.8)		
Intergenerational support							
Financial support						0.627	0.890
No	396 (37.2)	315 (79.5)	52 (13.1)	15 (3.8)	14 (3.5)		
Yes	669 (62.8)	533 (79.7)	94 (14.1)	23 (3.4)	19 (2.8)		
Emotional support						15.590	0.001
No	257 (24.1)	187 (72.8)	40 (15.6)	16 (6.2)	14 (5.4)		
Yes	808 (75.9)	661 (81.8)	106 (13.1)	22 (2.7)	19 (2.4)		
Instrumental support						17.459	0.001
No	551 (51.7)	466 (84.6)	57 (10.3)	14 (2.5)	14 (2.5)		
Yes	514 (48.3)	382 (74.3)	89 (17.3)	24 (4.7)	19 (3.7)		
ADL level						208.504	<0.001
Completely independent	895 (84.1)	760 (84.9)	100 (11.2)	23 (2.6)	12 (1.3)		
Mild impairment	114 (10.7)	76 (66.7)	24 (21.1)	7 (6.1)	7 (6.1)		
Moderate impairment	35 (3.3)	10 (28.6)	15 (42.9)	2 (5.7)	8 (22.9)		
Severe impairment	21 (1.9)	2 (9.5)	7 (33.3)	6 (28.6)	6 (28.6)		

#### 3.1.1 Multiple logistic regression model of intergenerational support, ADL, and risk level of psychological distress

Psychological distress was used as the dependent variable, and gender, age, household registration, marital status, education level, living arrangement, occupation, annual household income, intergenerational support, and ADL level were used as independent variables to establish an ordered multiple classification logistic regression model. The results (see Table 2) show that after controlling for demographic characteristics, household registration, annual household income, occupation, financial support, instrumental support, ADL, and the risk level of psychological distress in the elderly were significantly correlated (*p* < *0.05*). Compared with the elderly in rural areas, the elderly living in urban areas have a 38.4% lower risk of different levels of psychological distress (OR = 0.616, *p* < 0.01); the elderly with a household annual income of <30,000 RMB have a 1.699 times higher risk of different levels of psychological distress than the elderly with a household annual income of ≥30,000 RMB (OR = 1.699, *p* < 0.05); those who engaged in mental work before retirement had a 37.9% lower risk of different levels of psychological distress than those who engaged in manual work (OR = 0.621, *p* < 0.05); elderly people who had not received financial support from their children in the past year had a 1.476 times higher risk of different levels of psychological distress than those who had received financial support from their children (OR = 1.476, *p* < 0.05); compared with the elderly who received instrumental support from their children in the past year, the risk of different levels of psychological distress in the elderly who did not receive instrumental support from their children in the past year was reduced by 45.9% (OR = 0.541, *p* < 0.01); Among the elderly with severe, moderate and mild ADL impairment, the risk of different levels of psychological distress was 31.913 times higher (OR = 31.913, *p* < 0.001), 12.503 times higher (OR = 12.503, *p* < 0.001) and 2.901 times higher (OR = 2.901, *p* < 0.001) respectively compared with the elderly with complete ADL independence.

**Table 2:** Logistic regression with ordered multiple classification of intergenerational support, ADL, and psychological distress.

Variables	*N*	Model1			Model2		
		*β*	OR (95% CI)	*P*	*β*	OR (95% CI)	*P*
Sex							
Male	522				−0.208	0.812 (0.576–1.145)	0.235
Female	543				Reference	1.000	-
Age							
60–69 years	466				0.194	1.214 (0.745–1.978)	0.436
70–79 years	425				0.001	1.001 (0.621–1.614)	0.995
≥80 years	174				Reference	1.000	-
Area of residence							
Urban	381				−0.484	0.616 (0.427–0.889)	0.010*
Rural	684				Reference	1.000	-
Marital status							
Single	32				−0.214	0.807 (0.298–2.186)	0.673
Married	835				−0.287	0.751 (0.482–1.169)	0.205
Others	198				Reference	1.000	–
Educational level							
Primary school or below	676				−0.424	0.654 (0.261–1.645)	0.367
Middle school education	355				−0.459	0.632 (0.249–1.606)	0.335
Bachelor’s degree or above	34				Reference	1.000	-
Annual household income							
<30000Yuan	176				0.530	1.699 (1.115–2.588)	0.014*
≥30000Yuan	889				Reference	1.000	-
Occupation before retirement							
Mental work	244				−0.476	0.621 (0.404–0.956)	0.030*
Manual labor	821				Reference	1.000	-
Living situation							
Lives alone	157				−0.122	0.885 (0.552–1.418)	0.611
Living with others	908				Reference	1.000	–
Financial support							
No	396	0.307	1.359 (0.978–1.889)	0.068	0.389	1.476 (1.048–2.077)	0.026*
Yes	669	Reference	1.000	–	Reference	1.000	–
Emotional support							
No	257	0.374	1.454 (1.024–2.063)	0.036	0.365	1.441 (0.997–2.079)	0.052
Yes	808	Reference	1.000	–	Reference	1.000	–
Instrumental support							
No	551	−0.683	0.505 (0.366–0.697)	<0.001	−0.614	0.541 (0.381–0.769)	0.001**
Yes	514	Reference	1.000	-	Reference	1.000	–
ADL level							
Completely independent	21	3.419	30.539 (13.263–70.246)	<0.001	3.463	31.913 (13.491–75.415)	<0.001***
Mild impairment	35	2.575	13.131 (6.876–25.078)	<0.001	2.526	12.503 (6.398–24.435)	<0.001***
Moderate impairment	114	1.008	2.740 (1.782–4.212)	<0.001	1.065	2.901 (1.857–4.527)	<0.001***
Severe impairment	895	Reference	1.000	–	Reference	1.000	–

Model 1: unadjusted demographic characteristics; Model 2 adjusted for gender, age, household registration, marital status, education level, living arrangement, occupation, and annual household income; OR, Odds ratio; *P < 0.05; **P < 0.01; ***P < 0.001.

#### 3.1.2 Interaction between variables

**Figure 1:**
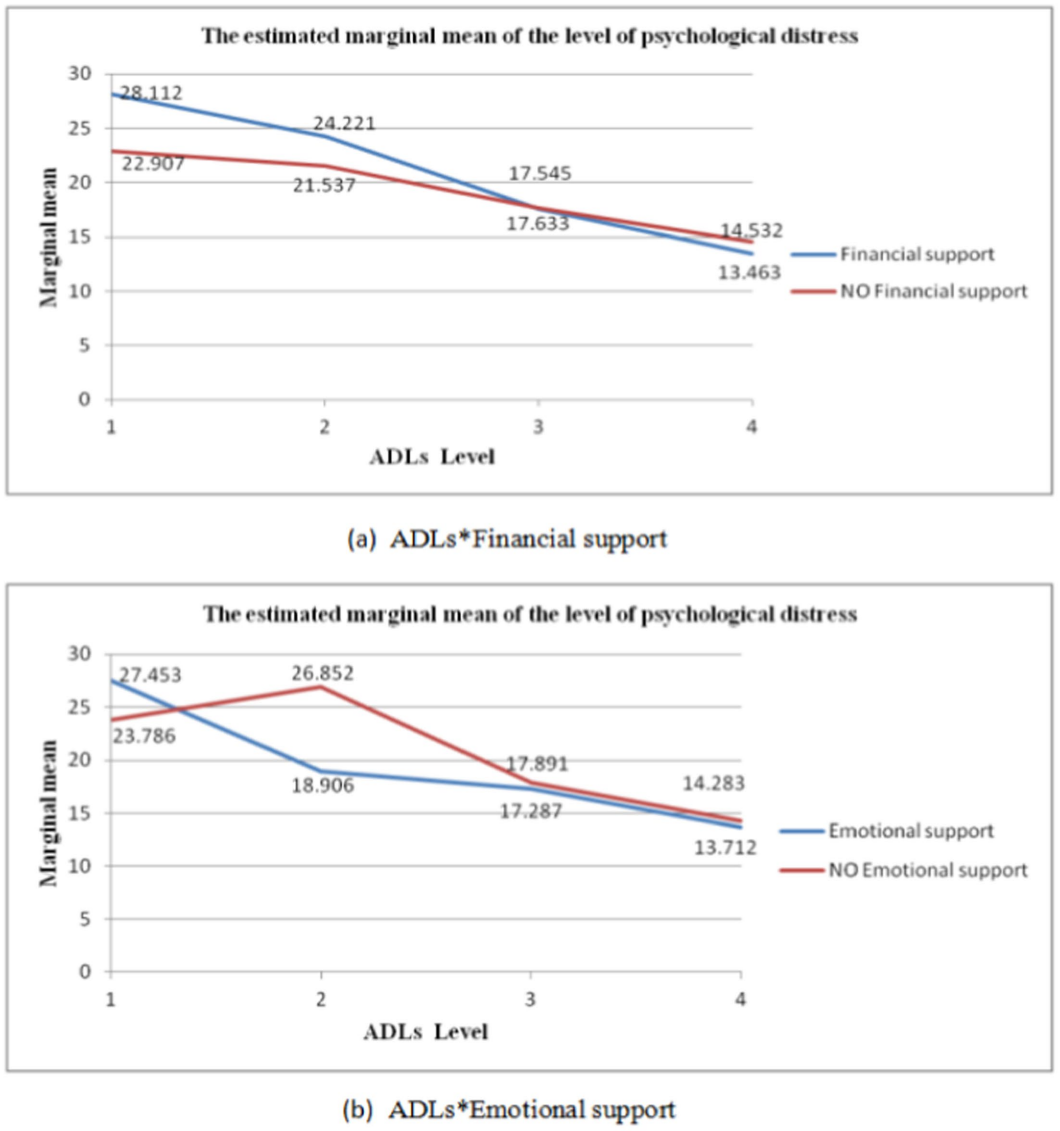
Second-order multiplicative interactions for ADL with intergenerational support. The horizontal axis represents the ADL level, and the vertical axis represents the marginal mean of psychological distress. The curves intersect or demonstrate a clear trend of intersection, indicating a significant interaction.

**Figure 2:**
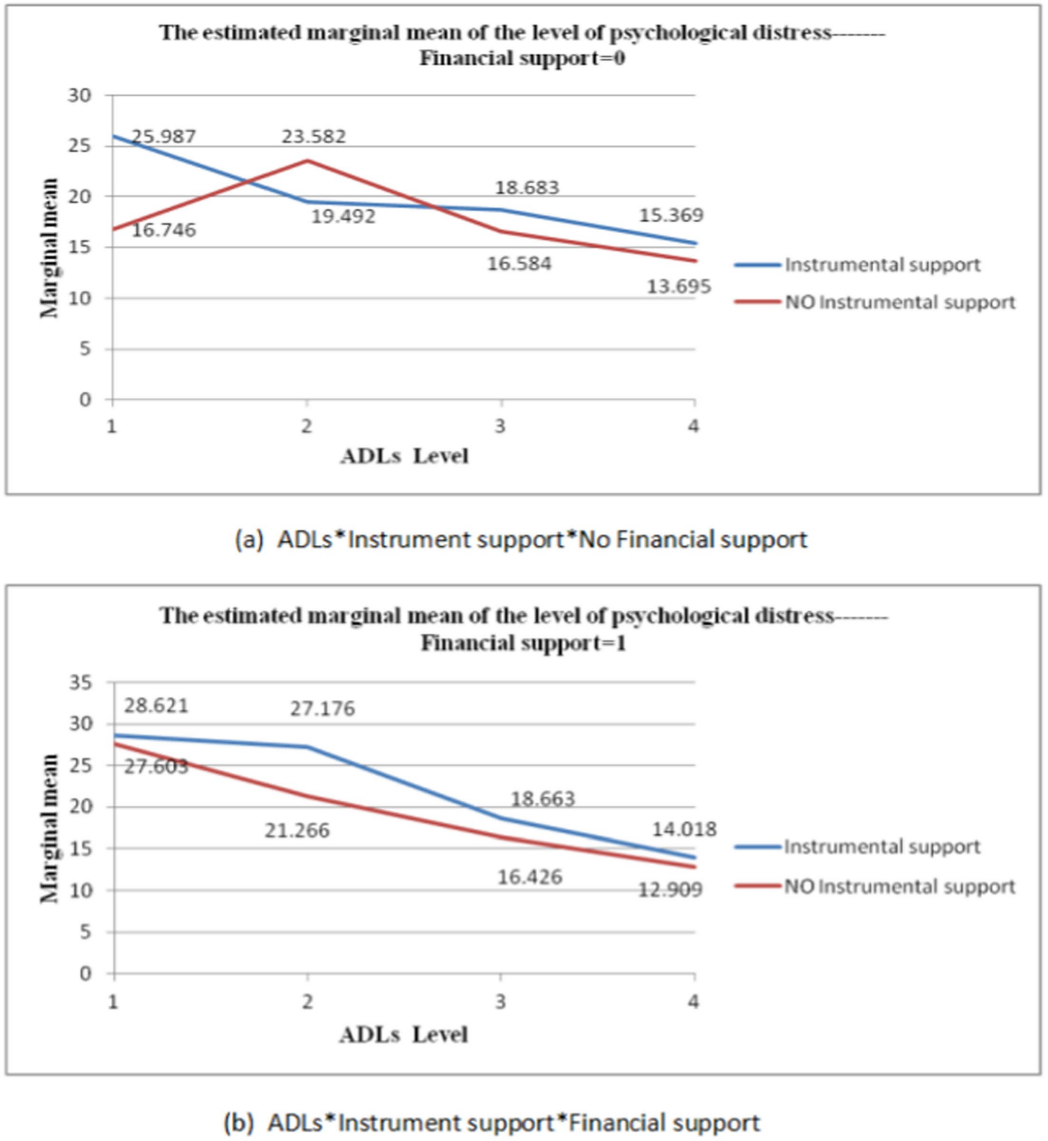
Third-order multiplicative interactions between ADL, Financial, support and Instrumental support. The horizontal axis represents the ADL level, and the vertical axis represents the marginal mean of psychological distress. The curves intersect or demonstrate a clear trend of intersection, indicating a significant interaction.

The dependent variable was psychological distress in the elderly, while the independent variables were ADL, intergenerational support, and their respective interaction terms. The results of the one-way analysis of variance, controlling for gender, age, household registration, marital status, educational level, living situation, occupation, and annual household income, are presented in Table 3. A significant interaction effect was observed between ADL and financial support (F = 221.570, *p* < 0.05) (see Figure 1A). Significant interaction effects were found between ADL and emotional support (F = 399.805, *p* < 0.01) (see Figure 1B). The interaction effects between ADL, financial support and instrumental support were found to be significant (F = 227.187, *p* < 0.05) (see Figures 2A,B).

**Table 3:** Testing of inter-subject effects on variables.

Variables	Adjusted *R*^2^	df	*F*	*P*
Sex	96.227	1	3.641	0.057
Age	40.103	1	1.517	0.218
Area of residence	81.367	1	3.079	0.030*
Marital status	15.820	1	0.599	0.439
Educational level	0.058	1	0.002	0.963
Occupation before retirement	136.669	1	5.171	0.023*
Annual household income	91.064	1	3.446	0.044*
Living arrangement	7.134	1	0.270	0.603
Financial support	108.556	1	4.107	0.043*
Emotional support	6.562	1	0.248	0.618
Instrumental support	163.737	1	6.195	0.013*
ADL	3107.244	3	39.189	<0.001***
Financial support * Emotional support	10.725	1	0.406	0.524
Financial support * Instrumental support	0.202	1	0.008	0.930
Emotional support * Instrumental support	0.432	3	0.016	0.898
ADL*Financial support	221.570	1	2.794	0.039*
ADL*Emotional support	399.805	3	5.042	0.002**
ADL*Instrumental support	70.651	3	0.891	0.445
Financial support * Emotional support * Instrumental support	51.238	1	1.939	0.164
ADL*Financial support * Emotional support	43.703	3	0.551	0.647
ADL*Financial support *Instrumental support	227.187	3	2.865	0.036*
ADL*Emotional support * Instrumental support	159.626	3	2.013	0.110
ADL*Financial support * Emotional support * Instrumental support	199.863	2	3.781	0.053

*P < 0.05; **P < 0.01; ***P < 0.001.

#### 3.1.3 The second-order interaction of ADL with intergenerational support

Table 4 illustrated that when the ADL level was completely independent, there was a mean difference of 1.068 (MD = −1.068, S.E. = 0.452, *p* < 0.05) between the elderly who received financial support from their children and those who did not. The interaction terms for the remaining levels were not statistically significant.

**Table 4:** The multiple interactions of ADL and financial support on psychological distress.

ADL	Financial support (I)	Financial support (J)	MD (I-J)	S.E.	*P*	95% CI
Severe impairment	Financial support	No financial support	5.205	3.064	0.090	(0.446, −11.219)
Moderate impairment	Financial support	No financial support	2.684	2.366	0.257	(−1.958, 7.327)
Mild impairment	Financial support	No financial support	−0.089	1.263	0.944	(−2.567, 2.390)
Completely independent	Financial support	No financial support	−1.068	0.452	0.018*	(−1.956, −0.181)

Adjusted for socio-demographic characteristics including gender, age, household registration, marital status, education level, living arrangement, occupation, and annual household income; S.E., Standard error; MD, Mean-diff; *p < 0.05.

As shown in Table 5, the more severe the ADL restriction, the higher the average level of psychological distress among the elderly. The average differences in psychological distress between the elderly with severe, moderate and mild ADL impairment who did not receive financial support from their children and the elderly who were completely independent were 8.375 (MD = 8.375, S.E. = 2.764, *p* < 0.01), 7.005 (MD = 7.005, S.E. = 2.103, *p* < 0.01) and 3.101 (MD = 3.101, S.E. = 1.170, *p* < 0.01). However, among the elderly who received financial support from their children, the average difference in psychological distress between the elderly with severe ADL impairment and moderately impaired, mildly impaired, and completely independent elders was 3.891 (MD = 3.891, S.E. = 1.784, *p* < 0.05), 10.568 (MD = 10.568, S.E. = 1.481, *p* < 0.001), 14.649 (MD = 14.649, S.E. = 1.390, *p* < 0.001); the mean differences in psychological distress between the elderly with moderate ADL impairment and the elderly with mild impairment and completely independent elderly were 6.676 (MD = 6.676, S.E. = 1.296, *p* < 0.001) and 10.757 (MD = 10.757, S.E. = 1.187, *p* < 0.001) respectively. The mean difference in psychological distress between the ADL level of mild impairment and completely independent elderly was 4.081 (MD = 4.081, S.E. = 0.652, *p* < 0.001). The interaction terms for other levels were not statistically significant.

**Table 5:** The multiple interactions of financial support and ADL on psychological distress.

Financial support	ADL (I)	ADL (J)	MD (I-J)	S.E.	*P*	95% CI
No Financial support	Severe impairment	Moderate impairment	1.370	3.431	0.690	(−5.362, 8.102)
		Mild impairment	5.273	2.951	0.074	(−0.518, 11.064)
		Completely independent	8.375	2.764	0.003**	(2.951, 13.799)
	Moderate impairment	Severe impairment	−1.370	3.431	0.690	(−8.102, 5.362)
		Mild impairment	3.903	2.350	0.097	(−0.709, 8.516)
		Completely independent	7.005	2.103	0.001**	(2.878, 11.131)
	Mild impairment	Severe impairment	−5.273	2.951	0.074	(−11.064, 0.518)
		Moderate impairment	−3.903	2.350	0.097	(−8.516, 0.709)
		Completely independent	3.101	1.170	0.008**	(0.806, 5.397)
	Completely independent	Severe impairment	−8.375	2.764	0.003**	(−13.799, −2.951)
		Moderate impairment	−7.005	2.103	0.001**	(−11.131, −2.878)
		Mild impairment	−3.101	1.170	0.008**	(−5.397, −0.806)
Financial support	Severe impairment	Moderate impairment	3.891	1.784	0.029*	(0.390, 7.393)
		Mild impairment	10.568	1.481	<0.001***	(7.661, 13.475)
		Completely independent	14.649	1.390	<0.001***	(11.921, 17.377)
	Moderate impairment	Severe impairment	−3.891	1.784	0.029*	(−7.393, −0.390)
		Mild impairment	6.676	1.296	<0.001***	(4.134, 9.219)
		Completely independent	10.757	1.187	<0.001***	(8.429, 13.086)
	Mild impairment	Severe impairment	−10.568	1.481	<0.001***	(−13.475, −7.661)
		Moderate impairment	−6.676	1.296	<0.001***	(−9.219, −4.134)
		Completely independent	4.081	0.652	<0.001***	(2.802, 5.360)
	Completely independent	Severe impairment	−14.649	1.390	<0.001***	(−17.377, −11.921)
		Moderate impairment	−10.757	1.187	<0.001***	(−13.086, −8.429)
		Mild impairment	−4.081	0.652	<0.001***	(−5.360, −2.802)

Adjusted for socio-demographic characteristics including gender, age, household registration, marital status, education level, living arrangement, occupation, and annual household income; S.E., Standard error; MD, Mean-diff; *P < 0.05; **P < 0.01; ***P < 0.001.

As shown in Table 6, when the ADL level was moderate, the average difference in psychological distress between the elderly who received emotional support from their children and those who did not was −7.947 (MD = −7.947, S.E. = 2.374, *p* < 0.05). The interaction terms for other levels were not statistically significant.

**Table 6:** The multiple interaction of ADL and emotional support on psychological distress.

ADL	Emotional support (I)	Emotional support (J)	MD (I-J)	S.E.	*P*	95% CI
Severe impairment	Emotional support	No Emotional support	3.667	2.797	0.190	(−1.820, 9.155)
Moderate impairment	Emotional support	No Emotional support	−7.947	2.374	0.001**	(−12.605, −3.288)
Mild impairment	Emotional support	No Emotional support	−0.603	1.266	0.634	(−3.088, 1.881)
Completely independent	Emotional support	No Emotional support	−0.571	0.452	0.207	(−1.458, 0.316)

Adjusted for socio-demographic characteristics including gender, age, household registration, marital status, education level, living arrangement, occupation, and annual household income; S.E., Standard error; MD, Mean-diff; *P < 0.05; **P < 0.01.

As shown in Table 7, among the elderly who received emotional support from their children, the more severely restricted their ADL, the higher average level of psychological distress among the elderly. The mean differences in psychological distress between the ADL levels of severe, moderate and mild impairment and the level of completely independent elderly people were 8.547 (MD = 8.547, S.E. = 2.294, *p* < 0.001), 10.166 (MD = 10.166, S. E. = 2.022, *p* < 0.001) and 13.741 (MD = 13.741, S.E. = 1.936, *p* < 0.001); the mean difference in psychological distress between the elderly with moderate impairment and those who were completely independent was 5.193 (MD = 5.193, S.E. = 1.269, *p* < 0.001); the mean difference in psychological distress between the elderly with mild impairment and the elderly who were completely independent was 3.575 (MD = 3.575, S.E. = 0.691, *p* < 0.001).

**Table 7:** The multiple interactions of emotional support and ADL on psychological distress.

Emotional support	ADL (I)	ADL (J)	MD (I-J)	S.E.	*P*	95% CI
No Emotional support	Severe impairment	Moderate impairment	−3.066	2.857	0.283	(−8.673, 2.540)
		Mild impairment	5.895	2.310	0.011*	(1.363, 10.427)
		Completely independent	9.503	2.079	<0.001***	(5.424, 13.581)
	Moderate impairment	Severe impairment	3.066	2.857	0.283	(−2.540, 8.673)
		Mild impairment	8.961	2.287	<0.001***	(4.473, 13.450)
		Completely independent	12.569	2.055	<0.001***	(8.537, 16.601)
	Mild impairment	Severe impairment	−5.895	2.310	0.011*	(−10.427, −1.363)
		Moderate impairment	−8.961	2.287	<0.001***	(−13.450, 4.473)
		Completely independent	3.607	1.150	0.002**	(1.352,5.863)
	Completely independent	Severe impairment	−9.503	2.079	<0.001***	(−13.581, −5.424)
		Moderate impairment	−12.569	2.055	<0.001***	(−16.601, −8.537)
		Mild impairment	−3.607	1.150	0.002**	(−5.863, −1.352)
Emotional support	Severe impairment	Moderate impairment	8.547	2.294	<0.001***	(4.045, 13.050)
		Mild impairment	10.166	2.022	<0.001***	(6.197, 14.134)
		Completely independent	13.741	1.936	<0.001***	(9.943, 17.539)
	Moderate impairment	Severe impairment	−8.547	2.294	<0.001***	(−13.050, −4.045)
		Mild impairment	1.618	1.415	0.253	(−1.157, 4.394)
		Completely independent	5.193	1.269	<0.001***	(2.703, 7.684)
	Mild impairment	Severe impairment	−10.166	2.022	<0.001***	(−14.134, −6.197)
		Moderate impairment	−1.618	1.415	0.253	(−4.394, 1.157)
		Completely independent	3.575	0.691	<0.001***	(2.220, 4.930)
	Completely independent	Severe impairment	−13.741	1.936	<0.001***	(−17.539, −9.943)
		Moderate impairment	−5.193	1.269	<0.001***	(−7.684, −2.703)
		Mild impairment	−3.575	0.691	<0.001***	(−4.930, −2.220)

Adjusted for socio-demographic characteristics including gender, age, household registration, marital status, education level, living arrangement, occupation, and annual household income; S.E., Standard error; MD, Mean-diff; *P < 0.05;**P < 0.01; ***P < 0.001.

Among the elderly who did not received emotional support from their children, the mean differences in psychological distress between the elderly with severely impaired ADL and those with mild impairment and completely independent elderly were 5.895 (MD = 5.895, S.E. = 2.310, *p* < 0.05) and 9.503 (MD = 9.503, S.E. = 2.079, *p* < 0.001) respectively; the mean differences in psychological distress between the elderly with moderate impairment and the elderly with mild impairment and completely independent elderly were 8.961 (MD = 8.961, S.E. = 2.287, *p* < 0.001) and 12.569 (MD = 12.569, S.E. = 2.055, *p* < 0.001) respectively; the mean difference in psychological distress between the elderly with mild impairment and those who were completely independent was 3.607 (MD = 3.607, S.E. = 1.150, *p* < 0.01). The interaction terms at other levels are not statistically significant.

#### 3.1.4 ADL and the multiplicative interaction of the third order of intergenerational support

As shown in Table 8, when the ADL level was moderate, the mean difference in psychological distress between the elderly who received both financial and instrumental support from their children and those who received only financial but not instrumental support was 5.910 (MD = 5.910, S.E. = 2.304, *p* < 0.05); when the ADL level was completely independent, the mean difference in psychological distress between elders who received both financial and instrumental support from their children and those who received only financial but not instrumental support was 1.110 (MD = 1.110, S.E. = 0.547, *p* < 0.05), while the mean difference between those who received only instrumental support and those who received neither financial nor instrumental support was 1.674 (MD = 1.674, S.E. = 0.735, *p* < 0.05). The interaction terms for the other levels were not statistically significant.

**Table 8:** ADL, financial support, and instrumental support for the three-stage interaction of psychological distress.

ADL	Financial support	Instrumental support (I)	Instrumental support (J)	MD (I-J)	S.E.	*P*	(95% CI)
Severe impairment	Financial support	Instrumental support	No instrumental support	1.018	2.705	0.707	(−4.290, 6.326)
	No financial support	Instrumental support	No instrumental support	9.242	5.162	0.074	(−0.888, 19.372)
Moderate impairment	Financial support	Instrumental support	No instrumental support	5.910	2.304	0.010*	(1.388, 10.431)
	No financial support	Instrumental support	No instrumental support	−4.090	4.155	0.325	(−12.243, 4.063)
Mild impairment	Financial support	Instrumental support	No instrumental support	2.236	1.196	0.062	(−0.111, 4.584)
	No financial support	Instrumental support	No instrumental support	2.099	2.234	0.348	(−2.285, 6.484)
Completely independent	Financial support	Instrumental support	No instrumental support	1.110	0.547	0.043*	(0.037, 2.182)
	No financial support	Instrumental support	No instrumental support	1.674	0.735	0.023*	(0.232, 3.116)

Adjusted for socio-demographic characteristics including gender, age, household registration, marital status, education level, living arrangement, occupation, and annual household income; S.E., Standard error; MD, Mean-diff; *P ≤ 0.05.

As shown in Table 9, when the ADL level was severe, the mean difference in psychological distress between those who received financial but not instrumental support and those who received neither financial nor instrumental support was 10.858 (MD = 10.858, S.E. = 4.235, *p* < 0.05); when the ADL level was moderate, the mean difference in psychological distress between the elderly who received both instrumental and financial support from their children and those who received only instrumental but not financial support was 7.684 (MD = 7.684, S.E. = 3.353, *p* < 0.05). When the ADL level was completely independent, the mean difference in psychological distress between the elderly who received both instrumental and financial support from their children and those who received only instrumental but not financial support was −1.351 (MD = −1.351, S.E. = 0.688, *p* ≤ 0.05). The interaction terms for the other levels were not statistically significant.

**Table 9:** The third-order multiplicative interactions of ADL, instrumental support, and financial support on psychological distress.

ADL	Instrumental support	Financial support (I)	Financial support (J)	MD (I-J)	S.E.	*P*	(95% CI)
Severe impairment	Instrumental support	Financial support	No financial support	2.634	4.013	0.512	(−5.240, 10.507)
	No instrumental support	Financial support	No financial support	10.858	4.235	0.011*	(2.513, 19.203)
Moderate impairment	Instrumental support	Financial support	No financial support	7.684	3.353	0.022*	(1.104, 14.264)
	No instrumental support	Financial support	No financial support	−2.316	3.342	0.489	(−8.875, 4.243)
Mild impairment	Instrumental support	Financial support	No financial support	−0.020	1.672	0.990	(−3.301, 3.361)
	No instrumental support	Financial support	No financial support	−0.157	1.896	0.934	(−3.878, 3.563)
Completely independent	Instrumental support	Financial support	No financial support	−1.351	0.688	0.050*	(−2.700, −0.001)
	No instrumental support	Financial support	No financial support	−0.786	0.583	0.178	(−1.930, 0.358)

Adjusted for socio-demographic characteristics including gender, age, household registration, marital status, education level, living arrangement, occupation, and annual household income; S.E., Standard error; MD, Mean-diff; *P ≤ 0.05.

As demonstrated in Table 10, among the elderly who did not receive either financial or instrumental support, the mean difference in psychological distress between those with moderate ADL impairment and older adults with mild impairment, who were completely independent, was 6.998 (MD = 6.998, S.E. = 3.413, *p* < 0.05), and 9.887 (MD = 9.887, S.E. = 3.008, *p* < 0.01) respectively. The interaction terms of other levels were found to be statistically insignificant.

**Table 10:** The third-order multiplicative interactions of financial support, instrumental support, and ADL on psychological distress.

Financial support	Instrumental support	ADL (I)	ADL (J)	MD (I-J)	S.E.	*P*	(95% CI)
No financial support	No instrumental support	Severe impairment	Moderate impairment	−6.836	4.716	0.147	(−16.090, 2.417)
			Mild impairment	0.162	4.025	0.968	(−7.736, 8.060)
			Completely independent	3.051	3.683	0.408	(−4.177, 10.278)
		Moderate impairment	Severe impairment	6.836	4.716	0.147	(−2.417, 16.09)
			Mild impairment	6.998	3.413	0.041*	(0.300, 13.696)
			Completely independent	9.887	3.008	0.001**	(3.985, 15.789)
		Mild impairment	Severe impairment	−0.162	4.025	0.968	(−8.060, 7.736)
			Moderate impairment	−6.998	3.413	0.041*	(−13.696, −0.300)
			Completely independent	2.889	1.732	0.096	(−0.510, 6.287)
		Completely independent	Severe impairment	−3.051	3.683	0.408	(−10.278, 4.177)
			Moderate impairment	−9.887	3.008	0.001**	(−15.789, −3.985)
			Mild impairment	−2.889	1.732	0.096	(−6.287, 0.510)
	Instrumental support	Severe impairment	Moderate impairment	6.496	4.659	0.164	(−2.647, 15.639)
			Mild impairment	7.304	3.934	0.064	(−0.416, 15.025)
			Completely independent	10.618	3.707	0.004**	(3.345, 17.892)
		Moderate impairment	Severe impairment	−6.496	4.659	0.164	(−15.639, 2.647)
			Mild impairment	0.809	3.235	0.803	(−5.540, 7.157)
			Completely independent	4.123	2.943	0.161	(−1.651, 9.897)
		Mild impairment	Severe impairment	−7.304	3.934	0.064	(−15.025, 0.416)
			Moderate impairment	−0.809	3.235	0.803	(−7.157, 5.540)
			Completely independent	3.314	1.572	0.035*	(0.229, 6.399)
		Completely independent	Severe impairment	−10.618	3.707	0.004**	(−17.892, −3.345)
			Moderate impairment	−4.123	2.943	0.161	(−9.897, 1.651)
			Mild impairment	−3.314	1.572	0.035*	(−6.399, −0.229)
Financial support	No instrumental support	Severe impairment	Moderate impairment	6.337	2.649	0.017*	(1.140, 11.535)
			Mild impairment	11.177	2.332	<0.001***	(6.601, 15.753)
			Completely independent	14.695	2.195	<0.001***	(10.387, 19.002)
		Moderate impairment	Severe impairment	−6.337	2.649	0.017*	(−11.535, −1.140)
			Mild impairment	4.840	1.768	0.006**	(1.370, 8.310)
			Completely independent	8.357	1.575	<0.001***	(5.268, 11.447)
		Mild impairment	Severe impairment	−11.177	2.332	<0.001***	(−15.753, −6.601)
			Moderate impairment	−4.840	1.768	0.006**	(−8.310, −1.370)
			Completely independent	3.518	0.965	<0.001***	(1.623, 5.412)
		Completely independent	Severe impairment	−14.695	2.195	<0.001***	(−19.002, −10.387)
			Moderate impairment	−8.357	1.575	<0.001***	(−11.447, −5.268)
			Mild impairment	−3.518	0.965	<0.001***	(−5.412, −1.623)
	Instrumental support	Severe impairment	Moderate impairment	1.446	2.373	0.543	(−3.211, 6.102)
			Mild impairment	9.958	1.815	<0.001***	(6.396, 13.521)
			Completely independent	14.603	1.684	<0.001***	(11.299, 17.906)
		Moderate impairment	Severe impairment	−1.446	2.373	0.543	(−6.102, 3.211)
			Mild impairment	8.513	1.892	<0.001***	(4.800, 12.226)
			Completely independent	13.157	1.766	<0.001***	(9.693, 16.622)
		Mild impairment	Severe impairment	−9.958	1.815	<0.001***	(−13.521, −6.396)
			Moderate impairment	−8.513	1.892	<0.001***	(−12.226, −4.800)
			Completely independent	4.644	0.874	<0.001***	(2.930, 6.359)
		Completely independent	Severe impairment	−14.603	1.684	<0.001***	(−17.906, −11.299)
			Moderate impairment	−13.157	1.766	<0.001***	(−16.622, −9.693)
			Mild impairment	−4.644	0.874	<0.001***	(−6.359, −2.930)

Adjusted for socio-demographic characteristics including gender, age, household registration, marital status, education level, living arrangement, occupation, and annual household income; S.E., Standard error; MD, Mean-diff; *P < 0.05; **P < 0.01; ***P < 0.001.

Among elders who received instrumental support but not financial support, the mean difference in psychological distress between elders with severe impairment, mild impairment, and those who were completely independent in ADL was 10.618 (MD = 10.618, S.E. = 3.707, *p* < 0.01), 3.314 (MD = 3.314, S.E. = 1.572, *p* < 0.05). The interaction terms of other levels were not found to be statistically significant.

Among the elderly who received financial support but not instrumental support, the mean differences in psychological distress between those whose ADL level was severe impairment and those whose ADL level was moderate impairment, mild impairment, and completely independent were 6.337 (MD = 6.337, S.E. = 2.649, *p* < 0.05), 11.177 (MD = 11.177, S.E. = 2.332, *p* < 0.001), 14.695 (MD = 14.695, S.E. = 2.195, *p* < 0.001) respectively; the mean difference between the elderly with moderate ADL impairment and elderly with mild ADL impairment, completely independent was 4.840 (MD = 4.840, S.E. = 1.768, *p* < 0.01), 8.357 (MD = 8.357, S.E. = 1.575, *p* < 0.001) respectively; the mean difference between the elderly with mild impairment in ADL and those who were completely independent was 3.518 (MD = 3.518, S.E. = 0.965, *p* < 0.001). The interaction terms for other levels were not statistically significant.

Among the elderly who received both financial and instrumental support from their children, the mean differences in psychological distress between those with severely impaired ADL and those with mild impairment, or those completely independent were 9.958 (MD = 9.958, S.E. = 1.815, *p* < 0.001) and 14.603 (MD = 14.603, S.E. = 1.684, *p* < 0.001) respectively; the mean difference between the elderly with moderately impaired ADL and those with mild impairment, or those completely independent, was 8.513 (MD = 8.513, S.E. = 1.892, *p* < 0.001), 13.157 (MD = 13.157, S.E. = 1.766, *p* < 0.001) respectively; the mean difference in psychological distress between the elderly with mild impairment in ADL and those who were completely independent was 4.644 (MD = 4.644, S.E. = 0.874, *p* < 0.001). The interaction terms for other levels were not statistically significant.

## 4 Discussion

In this study, an ordered multiple classification logistic regression model was constructed to analyze the effects of various factors, including ADL and intergenerational support, on psychological distress in the elderly. The results demonstrated that both ADL and intergenerational support were significantly associated with psychological distress in the elderly, which was consistent with previous research findings (Tang et al., 2022; Chao, 2012; Ormel et al., 2002). Based on these findings, an interaction model was established through one-way analysis of variance to further explore the effect of the interaction between ADL and intergenerational support on psychological distress in the elderly. These findings may assist in the identification of at-risk groups of older individuals with mental health concerns at an early stage, enabling the implementation of targeted interventions to enhance their mental wellbeing and provide empirical evidence for the advancement of healthy aging.

This study found that area of residence, annual household income, occupation, financial support, instrumental support, and ADL are significantly associated with psychological distress in the elderly. The risk of psychological distress among the elderly in urban areas was significantly lower than that in rural areas within the sample of this study. Considering that in urban areas with more adequate material and spiritual resources, older people have access to better medical resources and recreational places or facilities, which makes it easier for them to have their needs met, create positive and optimistic moods, and effectively prevent mental health problems such as depression or anxiety, and thus reduces the risk of psychological distress, this finding was consistent with the conclusions of previous studies (Zhang D. et al., 2022; Wu et al., 2021). Annual household income significantly affects the psychological distress of older people. A number of studies have indicated that individuals with higher incomes are less likely to experience depression, anxiety, or stress than those with lower incomes (Korda et al., 2014; Nagasu et al., 2019). Notably, the differences in occupation observed in this study are significantly negatively correlated with psychological distress in the elderly. The risk of psychological distress in the elderly was significantly lower for those engaged in mental work than for those engaged in manual work. A multitude of factors, including the elderly themselves, their families, and society at large, influence the mental health of the elderly. Consequently, it was imperative that families and society provide greater support and encouragement to this vulnerable population.

Both financial and instrumental support were significantly associated with psychological distress in older adults. In this study, the elderly who did not receive financial support from their adult children were found to be more likely to experience different levels of psychological distress. A number of studies have demonstrated that financial support from children not only addresses the daily needs and healthcare requirements of the elderly, but also fulfills certain filial expectations of the elderly (Xia et al., 2022; Lai et al., 2019; Wu et al., 2018). It could be posited that financial support from children could not only alleviate the psychological pressure associated with financial difficulties, but also serve as a means of care for their parents. In contrast to previous research findings (Wang et al., 2023), The risk of psychological distress was reduced among older adults who did not receive instrumental support from their children compared to those who received support. In other studies, the elderly were more likely to be the “givers” in intergenerational relationships, and they would experience frustration when they were unable to provide assistance to their offsprings (Birditt et al., 2012). The receipt of care from children would lead to feelings of self-doubt and a sense of being a burden to one’s offspring, which would have an adverse effect on mental health (Cheng, 2017). Interestingly, although the impact of emotional support from adult children on psychological distress in the elderly was not statistically significant in this study, interaction terms indicated that when ADL limitations were present, the risk of psychological distress in the elderly who received emotional support was lower than that of those who did not. Positive parent–child communication and a reciprocal balance between generations could facilitate the elderly’s perception of being needed, which in turn protects their self-esteem and reduces the occurrence of psychological distress.

The present study found a significant association between ADL and psychological distress in the elderly. This finding aligned with previous studies in this field (Xiao et al., 2022; Jeyagurunathan et al., 2017). In this study, more severe ADL limitations were associated with a higher risk of psychological distress in older adults. In comparison to individuals with normal activity function, those with ADL limitations were unable to complete daily life activities independently. This led to a long-term lack of confidence in solving problems independently, excessive dependence on the help of family members or informal caregivers, and over time, an increased likelihood of developing mental health problems such as stress, anxiety, and depression.

Furthermore, this study contributed to the existing literature by examining the impact of intergenerational emotional support on the mental health of the elderly (Jacobson et al., 2017; Li et al., 2019). While the effect of emotional support as an independent influencing factor on the psychological distress of the elderly was not significant, the interaction between ADL and intergenerational financial support, emotional support, and instrumental support had a significant effect on the psychological distress of elders.

Firstly, the second-order interaction between ADL and financial or emotional support demonstrated that when ADL was moderately impaired, the risk of psychological distress among the elderly who received emotional support from their children was significantly lower than that of the elderly who did not receive emotional intergenerational support. When the level of ADL was completely independent, financial support from children was beneficial in reducing psychological distress among the elderly. Furthermore, among the elderly who received financial or emotional support from their children, the average level of psychological distress decreased with the decrease in ADL impairment. Secondly, the results of the third-order interaction of ADL, financial support, and instrumental support indicated that among the elderly who received financial support from their children, those with extremely limited ADL levels exhibited a higher risk of psychological distress than those who did not receive financial support, regardless of whether their adult children provided instrumental support. Conversely, among the elderly who were completely independent in their ADL levels, those who only received financial support exhibited a significantly lower risk of psychological distress than those who received neither financial support nor instrumental support. Finally, the three-way interaction of financial support, instrumental support, and ADL demonstrated that among elders who received instrumental support from their children, the risk of psychological distress was higher than that of elders who did not receive instrumental support from their children, regardless of whether they received financial support. This risk was observed to increase with the degree of ADL restriction, which may correlate with the independence and self-esteem of the elderly. Many studies have shown that the elderly require more encouragement and a sense of identity (Abolfathi Momtaz et al., 2014; Zhang et al., 2023). Excessive instrumental support from their children might result in the elderly person believing that they require nursing and are no longer able to complete daily life tasks independently. Such circumstances would result in a lack of self-confidence among the elderly, as well as the onset of mental health problems such as anxiety and depression.

With the development of China’s economy and society, and the transformation of traditional family living patterns, more and more children now choose not to live with their parents any longer, but instead choose to help the elderly in financial and emotional ways. Nevertheless, some previous studies have shown that the elderly in China prefer to be supported by their families (Abolfathi Momtaz et al., 2014; Hu, 2019). In the context of the traditional home-based elderly care model still being dominant, the elderly’s care has to be taken by their adult children, as they are unable to complete daily tasks such as washing, eating and going to the toilet independently. The dilemma of needing to rely on others for help has caused the elderly to lose their self-confidence, believing that they cause a burden on their children. They have long-term feelings of guilt or self-blame, and then experience psychological distress such as anxiety and depression.

The findings of these studies provide empirical evidence to support the improvement of mental health in the elderly and the raising of their mental health level. When the elderly exhibit a low level of ADL restriction, it is imperative that they be provided with active financial and emotional support, that their material living standards be improved, and that they communicate more frequently with their children and other relatives. These measures are essential for effectively reducing the risk of psychological distress in the elderly. In the case of elderly individuals with a high level of ADL restriction, it is recommended that family members and society at large provide greater encouragement, engage in frequent communication, enhance self-confidence, alleviate psychological pressure, and thus reduce the risk of psychological distress.

Overall, when the level of ADL increases, it often stimulates an increase in the demand for medical services and dependence on the care of others. The total amount of intergenerational support required also changes accordingly. In the context of China’s filial piety culture, the traditional model of ageing at home still dominates, and adult children have always been the main providers of financial, emotional and instrumental support (Shu et al., 2021). However, restricted ADL and increased care requirements may lead to a deterioration in intergenerational relationships, with the elderly being unwilling to proactively express their needs for daily care, making their children think that they are a burden (Freitas et al., 2021; Jill Suitor et al., 2020). These phenomena show that as the ageing population increases, the issue of caring for the elderly requires the joint efforts of family members and society. China’s traditional culture of filial piety should continue to play a role, with children actively communicating, establishing good channels of intergenerational communication, encouraging the elderly to talk about their needs, and strengthening mutual support between generations. At the same time, society should provide more convenient social welfare programs such as community activities, chronic disease rehabilitation guidance, psychological counseling, and public health examinations to further improve the mental health of the elderly and add momentum to the realization of healthy ageing.

### 4.1 Limitations and further directions

Limitations of this study exist. Firstly, participants were surveyed through a scientific scale, yet the survey results were obtained from respondents’ memories, which may be subject to recall bias. Secondly, this was a cross-sectional study, and the dynamic trend of causal associations between ADL, intergenerational support and psychological distress over time could not be confirmed; therefore, in future studies, a longitudinal research design should be combined to test the causal relationships. Third, this study only utilized intergenerational support acquisition for older adults and did not consider the effects of “downward” intergenerational support giving on psychological distress in older adults; therefore, subsequent research should consider the effects of “downward” intergenerational support giving on psychological distress in older adults. Finally, long-term caregiving burdens can lead to deterioration of intergenerational relationships, and this study did not address the mental health status of family members’ caregivers, so the association between intergenerational support giving, older adults’ ADL limitations, and caregivers’ mental health statuses, such as depression and anxiety, should be examined in further research.

## 5 Conclusions and implications

The study is the first to combine intergenerational support and different levels of ADL to investigate the effectiveness of intergenerational “upwardly mobile” support situations and the combined effects of different levels of ADL on psychological distress in older adults.

First, our findings suggest that place of origin, average annual household income, nature of occupation, financial support, instrumental support, and ADL were significantly associated with psychological distress in older adults. In subsequent studies, all these factors may contribute to the early identification of direct or potential triggers of mental health problems in older adults, which will be crucial for achieving healthy aging and improving the mental health status of older adults.

Second, when ADL levels were moderate, older adults who received emotional support from their children had a significantly lower risk of psychological distress compared to those who did not receive emotional intergenerational support; and when ADL levels exhibited being completely independent, financial support from their children was beneficial in reducing psychological distress in older adults. The findings of this study point the direction of specific interventions to effectively reduce adverse mental health symptoms such as depression and anxiety in older adults and to improve the overall quality of life of older adults and society.

Finally, the risk of psychological distress among older adults who received instrumental support from their children increased with increasing ADL limitations, regardless of whether they received additional financial support. Such conclusion will play a guiding role in subsequent studies related to the care of disabled older adults and the maintenance of intergenerational family relationships, and further validates and complements the research in the field of older adults’ mental health, providing a new pathway option to improve older adults’ mental health, and enhancing the systematic and comprehensive nature of the research in the field.

## Data Availability

The raw data supporting the conclusions of this article will be made available by the authors, without undue reservation.
